# MiR-3664-3p through suppressing *ABCG2, CYP3A4, MCL1,* and *MLH1* increases the sensitivity of colorectal cancer cells to irinotecan

**DOI:** 10.1016/j.heliyon.2025.e41933

**Published:** 2025-01-15

**Authors:** Elham Farrokhnazar, Sahar Moghbelinejad, Reza Najafipour, Ladan Teimoori-Toolabi

**Affiliations:** a Research Institute for Prevention of Non-Communicable Diseases, Cellular and Molecular Research Center, Qazvin University of Medical Sciences, Qazvin, Iran; b Molecular Medicine Department, Biotechnology Research Center, Pasteur Institute of Iran, Iran; c Department of Molecular Medicine, Faculty of Medicine, Qazvin University of Medical Sciences, Qazvin, Iran; d Genetics Research Center, University of Social Welfare and Rehabilitation Science, Tehran, Iran

**Keywords:** Colorectal Neoplasm, Drug Resistance, Irinotecan, ATP Binding Cassette Transporter, Subfamily G, Member 2, Cytochrome P-450 CYP3A4, MCL1 protein, human, MutL Protein Homolog 1, microRNA, human

## Abstract

**Background:**

Colorectal cancer (CRC) is the third most frequently diagnosed malignancy worldwide. Currently, irinotecan (CPT-11) is used alone or in combination with other drugs to treat patients with advanced CRC. However, the 5-year survival rate for metastatic CRC remains below 10 %, largely due to chemotherapy resistance. Several genes, including *ABCG2, CYP3A4, MCL1*, and *MLH1* contribute to irinotecan resistance. This study aimed to identify microRNAs that simultaneously regulate the expression of these genes in irinotecan-resistant cell lines and study their effect on resistant colorectal cancer cells.

**Methods:**

Irinotecan-resistant colorectal cancer cell lines were developed by intermittently exposing HCT116 and SW480 cell lines to gradually increasing doses of irinotecan over four generations. These resistant cell lines were designated HCT116-R1, HCT116-R2, HCT116-R3, HCT116-R4 and SW480-R1, SW480-R2, SW480-R3, SW480-R4. The induction of resistance was confirmed using MTT assays, by calculating IC_50_ values for each generation and comparing them to the parental cells. The expression levels of the *ABCG2, CYP3A4, MCL1*, and *MLH1* genes, along with miR-3664-3p, were initially measured in all resistant and parental cell lines using quantitative real-time PCR. Following transfection of HCT116-R3 and SW480-R3 cells with pre-miR-3664-3p, the expression levels of *ABCG2, CYP3A4, MCL1, MLH1*, and miR-3664-3p were re-evaluated using real-time PCR.

**Results:**

In resistant cell lines derived from HCT116 and SW480, increased expression of the *ABCG2, CYP3A4*, and *MCL1* genes was observed. However, a reduction in *CYP3A4* expression was noted in the final resistant lines from both cell lines. Additionally, while *MLH1* expression increased in HCT116-derived cell lines, no significant increase was observed in SW480-derived lines. A consistent decrease in miR-3664-3p expression was found across all resistant cell lines. When we transfected HCT116-R3 and SW480-R3 cells with pre-miR-3664-3p, there was an increase in miR-3664-3p expression and a reduction in *ABCG2, CYP3A4, MCL1*, and *MLH1* gene expression. This led to increased sensitivity to irinotecan.

**Conclusion:**

It can be concluded that miR-3664-3p can be considered a regulator of resistance to irinotecan by modulating the expression of *ABCG2, CYP3A4, MCL1*, and *MLH1* genes.

## Graphical abstract

**Figure:**
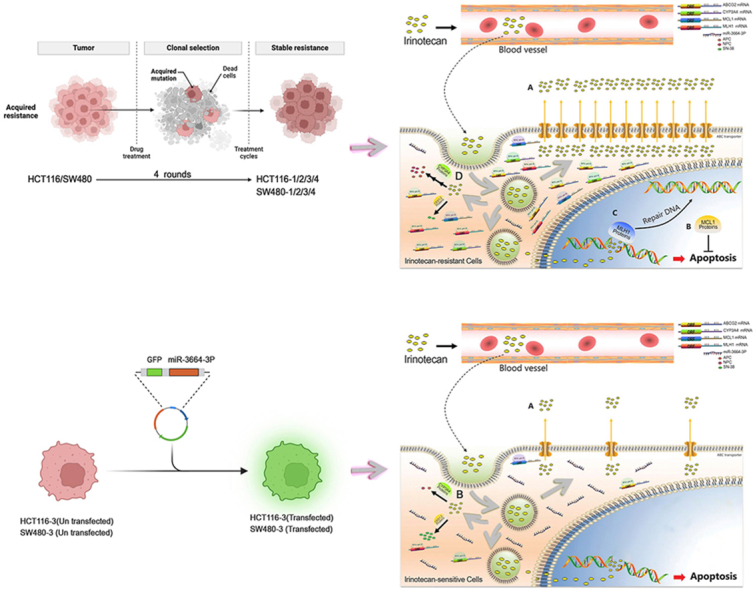

## 1 Introduction

Colorectal cancer (CRC) is the third most commonly diagnosed cancer and the second leading cause of cancer-related mortality worldwide [1,2]. In 2020, about 1.9 million individuals were diagnosed with colorectal cancer. While the disease is more prevalent among older individuals and has historically been more common in developed Western countries, recent years have seen a significant increase in cases in less developed countries and among younger generations in both developed and developing countries [3]. Approximately 20 % of patients diagnosed with colorectal cancer (CRC) present with metastases at the time of diagnosis. Additionally, up to 50 % of individuals initially diagnosed with a localized illness will eventually experience metastasis. CRC can spread through lymphatic and blood routes and contiguous in the peritoneum [4]. The most common sites for metastasis include regional lymph nodes, the liver, lungs, and peritoneum [2].

Despite advancements in therapy, the number of patients with metastatic colorectal cancer (mCRC) is rising due to therapy resistance, which is often caused by a small subset of cancer cells [1]. Improving the outlook of patients with metastatic colorectal cancer (mCRC) and locally advanced rectal cancer (LARC) remains challenging.

The five-year survival rate for mCRC patients is lower than 15 % indicating a poor prognosis [5]. Despite significant advancements in treatment options, conventional chemotherapy remains the primary choice, particularly for tumors that have metastasized [4].

Irinotecan is commonly used as the first-line therapy for colorectal cancer. It is an analog of camptothecin and works by inhibiting topoisomerase I, which disrupts transcription and DNA replication. Irinotecan can be administered alone or in combination with 5-fluorouracil (5-FU) and folic acid (FA). Combination chemotherapy regimens have proven to be more effective than single-agent chemotherapy [6]. However, chemotherapy resistance poses a significant challenge in effectively treating cancer [7]. Studies indicate that more than 90 % of patients with metastatic cancer undergo multiple rounds of chemotherapy due to drug resistance [8]. As a result, identifying agents that can reduce chemotherapy resistance in cancer cells is crucial [7].

Cancer cells can develop resistance to anticancer drugs due to various factors. Individual genetic variations in tumor cells and acquired resistance mechanisms are key factors. Acquired resistance mechanisms can be multi-drug resistance which is often caused by hyperactivity of ATP-binding cassette (ABC) transporters, suppression of apoptosis (cell death), and alterations in drug metabolism. Mechanisms of alterations in these genes include epigenetic modifications affecting drug targets, enhanced DNA repair process, and gene amplification. These elements play an instrumental role in the formation of resistance [8].

ABCG2, often referred to as the breast cancer resistance protein (BCRP), is recognized as a key transporter associated with drug absorption and elimination processes. It belongs to the ATP-binding cassette (ABC) transporter family [9]. Abnormal expression of this protein has been observed in various tissues, including the intestinal epithelium, placenta, blood-brain barrier, and different types of stem cells [9]. BCRP plays a crucial role in expelling a variety of anticancer drugs from cells, including quinazoline-based ErbB1 inhibitors, methotrexate, flavopiridol, camptothecin-derived and indolocarbazole topoisomerase I inhibitors, and mitoxantrone [10].

This export capability provided by the ABC transporter, leads to multidrug resistance in cancer cells. When the ABC transporter is highly expressed, these cells can effectively expel conventional chemotherapeutic drugs, which diminishes the effectiveness of treatment. Research has shown that ABCG2 is upregulated in various cancer cell lines with drug resistance, including gastric, breast, colon, lung, and ovarian cancers that exhibit drug resistance. This upregulation plays a significant role in resistance to cytotoxic compounds such as mitoxantrone and SN-38, the active metabolite of irinotecan. Patients with elevated *ABCG2* expression typically show poor responses to chemotherapy, suggesting that tumors with high levels of *ABCG2* are more likely to develop drug resistance. Additionally, increased *ABCG2* expression has also been identified in irinotecan-resistant colorectal cancer (CRC) cell lines and metastatic patients undergoing treatment with irinotecan. Therefore, heightened ABCG2 activity is considered a critical factor in driving chemotherapy resistance, metastasis, and disease recurrence. As a result, blocking the function of the ABCG2 pump may be a promising strategy to overcome chemotherapy resistance [11,12]. Cytochrome P450 3A4 (CYP3A4) is a key enzyme in the cytochrome P450 subfamily, playing a vital role in the metabolism of over half of all therapeutic drugs. It is primarily found in the intestine and liver, which are crucial organs for the oxidation and elimination of drugs. CYP3A4 can metabolize a wide range of approved anticancer drugs, thanks to its ability to process diverse chemical and molecular structures and variations in the mechanisms of action.

These anticancer drugs include antiestrogens, nitrogen mustards, taxanes, topoisomerase inhibitors (such as irinotecan), vinca alkaloids, as well as tyrosine kinase inhibitors (TKIs) like sorafenib and imatinib. The function and toxicity of a drug and its metabolites are significantly influenced by the activity of the enzyme CYP3A4. For instance, if the original compound is a prodrug, alterations in its metabolism-due to inhibition or induction-can lead to a decrease or an increase in its therapeutic effectiveness and side effects. Conversely, if the parent compound is more potent than its metabolite. Changes in metabolism can impact the drug's exposure, thereby altering its beneficial or harmful effects. Taking irinotecan as a specific example, it is a prodrug that undergoes two main metabolic pathways. The primary path involves carboxylesterase, which converts irinotecan into SN-38. This metabolite, SN-38 exhibits an anticancer effect that is 100–1000 times stronger than that of the original compound. Additionally, in the liver, irinotecan is metabolized by CYP3A4/5 leading to the formation of metabolites known as APC and NPC. This oxidation process results in inactive byproducts such as 7-ethyl-10-[4-amino-1-piperidino]carbonyloxy camptothecin (NPC) and 7-ethyl-10-[4-N-(5-amino pentanoic acid)-1-piperidino]-carbonyloxycamptothecin (APC). When CYP3A4 is highly active, it can significantly diminish the production of the active form of irinotecan, SN-38(12). Research has demonstrated that increased expression of *CYP3A4* mRNA in tumor tissues can accelerate drug metabolism, potentially leading to reduced efficacy or even resistance to the treatment [13]. Myeloid cell leukemia 1 (MCL1) is a protein that belongs to the BCL-2 family which plays a critical role in maintaining mitochondrial integrity-a key factor for cell survival. MCL1 is essential for various cell types' survival, differentiation, and development. However, it is found to be excessively overexpressed in many human cancers. MCL1 functions by binding to the BH3 domains of pro-apoptotic protein within the BCL-2 family, effectively preventing caspase activation and mitochondrial outer membrane permeabilization (MOMP), which in turn helps avert cell death. Recent studies have highlighted the significant role of MCL1 in tumor initiation, cancer cell survival, and resistance to multiple anti-cancer treatments. Senescence refers to a state in which cells cease to divide and undergo morphological and biochemical changes [14]. Numerous studies in both humans and animals suggest that senescence can limit tumor growth and improve the clinical outcome [15,16]. As a result, targeting senescence has become an important strategy for chemotherapy drugs [15]. Recent research indicates that MCL1 is essential in inhibiting chemotherapy-induced senescence (CIS). When *MCL1* is over-expressed, it prevents CIS, whereas the downregulation of *MCL1* increases cell sensitivity to CIS. Interestingly, the MCL1 component, responsible for preventing senescence, is distinct from one that inhibits apoptosis. Studies conducted in living organisms have demonstrated that reducing the amount of MCL1 level can significantly slow tumor growth. Regardless of p53 expression, overexpressing *MCL1* in cells with abundant p53 makes them resistant to CIS and promotes tumor growth [16]. Approximately 10–20 % of colorectal cancer (CRC) cases exhibit mutations or abnormal expression in mismatch repair (MMR) genes, including *Pms2*, *MLH1*, *Msh2*, and *Msh6*. Defects in MMR can be identified through Microsatellite Instability (MSI) analysis [17]. Sporadic cancers associated with hyper-methylation of the MutL homolog-1 (*MLH1*) promoter account for nearly 30 % of MSI CRCs, while others involve a hereditary MMR defect known as Hereditary Non-Polyposis Colorectal Cancer (HNPCC) or Lynch syndrome [18]. As a result, MSI status has become a critical biomarker in the metastatic setting for defining therapeutic options [19]. CRCs deficient in *MLH1* and exhibiting microsatellite instability (MSI) tend to be less invasive compared to *MLH1*-proficient cancers. Research by Manzoor et al. found that *MLH1*-proficient cells showed reduced sensitivity to the cytotoxic effects of 5-FU, irinotecan, and doxorubicin, a finding supported by subsequent studies. The important role of *MLH1* in facilitating chemo-resistance and promoting cancer cell survival is attributed to its ability to increase LC3 levels and induce nucleophagy in the cells treated with 5-FU and irinotecan [20].

The PI3K/AKT signaling pathway is a common molecular route that links the *ABCG2*, *CYP3A4*, and *MCL1* genes, particularly in the contexts of cancer and drug resistance [[21], [22], [23]]. ABCG2 functions as a drug efflux pump, contributing to multidrug resistance by expelling chemotherapy agents from cancer cells. This gene is upregulated by PI3K/AKT signaling [21]. CYP3A4, a major enzyme that contributes to metabolizing a broad spectrum of xenobiotics, such as drugs, can also contribute to drug resistance [22]. Its expression is influenced by PI3K/AKT signaling. The anti-apoptotic gene *MCL1* is similarly upregulated by PI3K/AKT signaling, promoting cell survival and contributing to treatment resistance [23]. Although no specific pathway has been linked to the increased level of *MLH1*, pathways that support DNA repair or epigenetic stability may contribute to maintaining or indirectly influencing *MLH1* levels [24].

MicroRNAs (miRNAs) are short, non-coding RNA molecules composed of 19–24 nucleotides. They primarily function by binding to the 3′ untranslated regions (3’ UTRs) of their target mRNAs, which regulates gene expression [25]. This regulation mainly occurs using two pathways: mRNA degradation and translation inhibition. In mRNA degradation, high complementarity between the miRNA and the mRNA attracts enzymes that degrade the mRNA, reducing its levels. In contrast, translation inhibition takes place when there is partial complementarity preventing ribosomes from efficiently translating the mRNA. This activity of miRNAs enables precise control over gene expression [26].

Studies suggest that approximately 2000 miRNAs regulate over half of all protein-coding genes. These small molecules significantly influence various signaling pathways that affect functions such as proliferation, differentiation, migration, cell cycle progression, apoptosis, carcinogenesis, and drug metabolism. Moreover, they play a role in mediating resistance to various anti-cancer medications. MicroRNAs (miRNAs) have emerged as a promising therapeutic approach for personalized treatment, aiming to enhance drug effectiveness or modulate patient responses to therapies [25]. The role of miRNAs in regulating gene expression is well established, and it is evident that these small molecules also influence drug performance through various mechanisms [26]. For example, overexpression of miR-302c-5p decreases the expression of *Abcb1*, leading to increased sensitivity to oxaliplatin [27]. In non-small cell lung cancer (NSCLC), heightened expression of miR-218 promotes apoptosis and causes cell cycle arrest at the G0/S checkpoint, enhancing sensitivity to cisplatin by downregulating *Runx2* [28]. In cervical cancer, downregulation of miR-499a enhances the anticancer properties of cisplatin by inhibiting proliferation, colony formation, apoptosis resistance, cell cycle progression, migration, and invasion. Additionally, overexpression of miR-519c increases sensitivity to irinotecan by reducing *ABCG2* expression [29]. MiRNA molecules are crucial in regulating gene expression in cancer cells. For example, overexpression of miR-17 reduces PTEN levels, which can lead to resistance against oxaliplatin, irinotecan, and 5-FU chemotherapy, ultimately resulting in lower survival rates [30]. Conversely, miR-3664-5p is downregulated in gastric cancer tissues and is positively associated with patient prognosis. Increased levels of this miRNA suppress the proliferation and metastasis of gastric cancer cells by targeting metadherin (MTDH) and inhibiting the NF-κB signaling pathway [31]. Furthermore, in two colon cancer cell lines, miR-3664-5p targets *Abcb1* and *Gstp1*. Downregulation of this miRNA increases resistance to oxaliplatin chemotherapy [27]. Our research aimed to investigate the effects of targeting drug-resistance genes (*ABCG2, CYP3A4*, *MCL1,* and *MLH1*) using specific miRNAs to enhance sensitivity to irinotecan. Through in silico methods, we identified a miRNA that can simultaneously target all four genes. Subsequently, we developed irinotecan-resistant colorectal cancer cell lines from HCT116 and SW480 cells. Finally, we examined the expression levels of both the target genes and miR-3664-3p.

## 2 Materials and methods

### 2.1 Cell culture

The SW480 cell line was obtained from the National Cell Bank of Iran (Pasteur Institute of Iran, Tehran, Iran) after its initial purchase from ATCC (Catalog No.: CCL-228). The HCT116 cell line was sourced directly from the American Type Culture Collection (Manassas, VA, USA; Catalog No.: CCL-247). Both cell lines were screened and verified to be mycoplasma-free and were authenticated by STR analysis one year before the start of this project. Cells were cultured in an incubator at 37 °C with 5 % CO₂ in high-glucose Dulbecco's Modified Eagle's Medium (DMEM) supplemented with 10 % heat-inactivated fetal bovine serum (FBS), 2 mM l-glutamine, 100 units/mL penicillin, and 100 μg/mL streptomycin. The culture medium was refreshed every two days, and molecular testing confirmed that the cell lines remained mycoplasma-free before further experimentation.

### 2.2 Establishment of irinotecan-resistant HCT116 and SW480 cell lines

Initially, the IC₅₀ (half-maximal inhibitory concentration) values for the HCT116 and SW480 cell lines in response to irinotecan were determined using the MTT assay. After establishing the IC₅₀ for each cell line, cells were treated with progressively increasing concentrations of irinotecan. The initial dose was set at 80 % of the calculated IC₅₀ for the parental cell lines. The drug-containing medium in each flask was refreshed every four days, with irinotecan added at a dose corresponding to 80 % of the calculated IC₅₀ from the previous generation, and was added to the cell culture along with the fresh medium. This process continued for two weeks. Ultimately, only a small number of cells remained, capable of surviving and adapting to the cytotoxic effects of irinotecan, thereby developing drug resistance. Following the treatment period, the medium was replaced with fresh medium lacking irinotecan but enriched with non-essential amino acids (0.1 mM) (GIBCO, USA) and L-glutamine (2 mM) for 5–6 weeks. This medium enrichment promoted the proliferation and expansion of the adapted cells, resulting in the first generation of irinotecan-resistant cell lines. The same procedure was repeated four times for the HCT116 and SW480 cell lines. The process continued until no cells could tolerate the drug (80 % of the calculated IC₅₀ from the previous generation), and no viable cells were observed two months after discontinuing irinotecan treatment. With each iteration, the cells became increasingly resistant to higher drug concentrations compared to the previous generation.

The drug was diluted to a concentration of 1/10 using a stock solution of 20 mg/mL. Irinotecan was used to treat the HCT116 cell line at concentrations of 24, 44, 107.2, and 232 ng/μL. To generate resistant cells from SW480, the concentrations were 24, 48, 64, and 176 ng/μL.

These concentrations were selected based on 80 % of the IC₅₀ values calculated from the previous generation. The cell lines derived from HCT116 after four rounds of resistance induction were named HCT116-R1, HCT116-R2, HCT116-R3, and HCT116-R4. Similarly, the SW480-derived cell lines after four rounds of resistance induction were named SW480-R1, SW480-R2, SW480-R3, and SW480-R4. This method has been employed in previous studies [27,[32], [33]].

### 2.3 Cell viability assay by MTT

The MTT assay was utilized to evaluate the cytotoxic effects of irinotecan. For this purpose, (4, 5-dimethylthiazol-2-yl)-2, 5-diphenyl-tetrazolium bromide (Sigma, Germany) was employed.

Initially, SW480 and HCT116 cell lines were seeded at a density of approximately 2 × 10⁴ cells per well and cultured in 96-well plates for 24 h under standard conditions (5 % CO₂ atmosphere, 37 °C temperature, and 95 % relative humidity). After this 24-h incubation in a CO₂ incubator, the cells were exposed to varying doses of irinotecan to determine their IC₅₀ values. The cells were then incubated for an additional 72 h. Following this incubation period, the wells were washed with PBS, and a new medium (supplemented with 10 % FBS) was added along with 10 % MTT solution (prepared fresh at 5 mg/mL in PBS). The plate was incubated for 4 h at 37 °C in a 5 % CO₂ atmosphere. Afterward, the medium was discarded, and 100 μL of isopropanol was added to the wells. The formazan crystals were then dissolved by shaking the plate. Finally, the absorbance of the treated cells was recorded at 570 nm using an ELISA reader, with 630 nm as the reference wavelength. The optical density of the treated cells was adjusted relative to that of the untreated cells. Each experiment was conducted in triplicate. IC₅₀ values were determined utilizing the 8.0.2 version of the GraphPad Prism program (GraphPad program, La Jolla, CA, USA) by plotting the measured cell viability (%) concerning irinotecan levels.

### 2.4 Predicting targeting miRNAs

Previous studies have shown that *ABCG2, CYP3A4, MCL1*, and *MLH1* genes are associated with the development of resistance to irinotecan in colorectal cancer [13,16,20,[34], [35], [36], [37], [38]]. To find miRNAs targeting these genes bioinformatics resources were utilized to identify a microRNA that targets all four genes simultaneously. These microRNAs can reduce their expression by binding to the 3′ untranslated region (3’ UTR). The impact of this microRNA on decreasing drug resistance in cancer cells was subsequently investigated.

Initially, two criteria were applied for selecting a miRNA. First, each selected miRNA was required to target all four genes. Second, this targeting needed to be confirmed by multiple databases. The online databases used in this study consisted of TargetScan (http://www.targetscan.org/), miRWalk (http://mirwalk.umm.uni-heidelberg.de/), miRDB (http://www.mirdb.org/miRDB/download.html), and Diana (http://diana.imis.athena-innovation.gr/). Each database used different predictive algorithms. To make a selection within the identified miRNAs, various features were assessed in the aforementioned databases. These features included the seed match between mRNAs and miRNAs, the cross-species conservation, the thermodynamic properties of miRNA-mRNA duplexes, the sequences, and the scores provided by the databases. Although miRNAs, unlike designed siRNAs, can target multiple genes, we investigated additional targets of the selected miRNAs to determine whether they influence important genes in other signaling pathways (Supplementary File 1). MiRNAs that targeted higher-scoring genes from other pathways were excluded from our selection. It is essential to validate all predicted targets of miRNAs through further in vitro studies.

To ensure accurate selection, interactions between miRNAs and the 3′ UTR regions of the target genes were assessed, along with analyzing their free energy characteristics. First, the sequences of the 3′UTR regions were identified from the NCBI database. Then, the sequences of miRNAs were obtained from the miRBase database (http://mirtarbase.mbc.nctu.edu.tw/). These miRNA and 3′UTR sequences were then entered into the RNAhybrid website (https://bio.tools/rnahybrid). Finally, the interactions between the miRNAs and the 3′UTR regions of the *ABCG2, CYP3A4, MCL1*, and *MLH1* genes were analyzed.

To validate the prediction parameters, two additional websites were used: https://bio.tools/rnahybrid and http://cm.jefferson.edu/rna22v1.0/. These sites calculated Gibbs free energy changes (ΔG), based on entropy changes (ΔS) and enthalpy changes (ΔH) within system. A reaction was deemed thermodynamically feasible when there was a negative change in energy, indicating an increase in the system's stability. This means that the enthalpy changes had to be negative. The formula relating enthalpy to free energy and temperature was applied to determine this. Finally, based on a comparison of the results from all the aforementioned analyses, miR-3664-3p was selected as the best miRNA.

### 2.5 Reverse transcription and real-time q-PCR

#### 2.5.1 Stem-loop and primers design

A universal primer and probe were used to measure miRNA levels. Stem-loop primers were designed based on previous studies [27]. To facilitate efficient binding of the stem-loops to the target miRNA, mature miRNA sequences were retrieved from the miRBase and NCBI databases. Sequences of the *ABCG2*, *CYP3A4*, *MCL1*, and *MLH1* genes were retrieved from the NCBI database to measure RNA levels. The NCBI Primer-BLAST tool designed forward and reverse primers for these genes. The primers for both miRNA and RNA were further evaluated using tools such as Gene Runner, Oligo 7, the IDT website (https://www.idtdna.com/), and Primer-BLAST. These evaluations included assessments of melting temperature, secondary structure, ΔG, and alignment with the template sequences.

#### 2.5.2 RNA isolation and cDNA synthesis

Initially, SW480 and HCT116 cell lines were seeded at a density of approximately 4 × 10^5^ cells per well of the six-well plate. When the cells reached 80 % confluency were detached from the bottom of the flask using 1 mL of trypsin and then centrifuged at 1500 rpm for 5 min. The supernatant was discarded, and 1 mL of PBS was added. Following another centrifugation step, the supernatant was removed, and the cell pellet was used for total RNA extraction using the Trizol reagent technique. Specifically, 800 μL of Trizol (Invitrogen) was added to the cells and incubated for 5 min at room temperature. Next, 200 μL of chloroform was added, and the mixture was vortexed vigorously for 15 s before being incubated at room temperature for 2–3 min. The samples were then centrifuged at 12,000 rpm for 10 min resulting in the formation of three distinct phases. Approximately 90 % of the upper aqueous phase was transferred to a new microcentrifuge tube, and an equal volume of isopropanol was introduced. The mixture was incubated at −20 °C for 30 min and centrifuged at 12,000 g. After removing the supernatant, 500 μL of 75 % ethanol was added to the RNA pellet, and the samples were centrifuged once more at 12,000 g at 4 °C. Finally, the RNA pellet was re-dissolved in 50 μL of DEPC-treated water.

The miRNA extraction protocol was similar to the RNA extraction process, except incubating the isopropanol eluates at −20 °C overnight and subsequent centrifugation at 12,000 rpm at 4 °C. The quality of the extracted miRNA was assessed by determining the A260/A280 ratio.

The procedure for synthesizing cDNA from miRNA and mRNA is outlined below: to synthesize cDNA from miRNA, 2 μg of extracted miRNA and 50 nM of stem-loop primers were transferred to a tube and heated to 65 °C for 10 min. The tubes were then placed on ice. Next, 1 μl of AddScript Enzyme Solution (Addbio, Korea), 1 mM of dNTP mixture (Addbio, Korea), and 10 μl of reaction buffer (Addbio, Korea) were added to the tube and incubated at 50 °C for 60 min. The mixture was subsequently heated to 80 °C for 5 min.

The same procedure was followed as above for synthesizing cDNA from mRNA, but the stem-loop primers were replaced with 10 nM of oligo dT. All cDNA synthesis was conducted using a PeqSTAR96X Universal thermocycler (Germany). Once synthesized, the cDNA was stored at −20 °C until needed.

#### 2.5.3 Quantitative real-time PCR

The StepOne-Plus™ Real-Time PCR System was used to conduct quantitative real-time PCR to measure mRNA levels of various genes. SYBR Green qRT-PCR (Ampliqon RealQ plus 2 Master Mix) was employed to assess gene expression gene expression. The sequences of the primers are provided in Table 1. Each reaction mixture consisted of 50 ng cDNA, 10 μl of SYBR Green I Master Mix, and 5–10 pmol of each primer. The thermal cycling protocol included enzyme activation at 95 °C for 15 min, followed by 40 cycles of denaturation at 95 °C for 20 s, annealing at 60 °C for 30 s, and extension at 72 °C for 30 s. The reference gene used for the normalization of gene expression was *Gapdh*. The specificity of the amplified sections was verified through melting curve analysis.

**Table 1:** Sequence of primers and stem-loop.

Name of the gene	Sequence of oligonucleotides (5′–3′)
*ABCG2-*F	CACGACATGGATTGGCATTG
*ABCG2*-R	CGATGCCCTCTTTCCAC
*CYP3A4*-F	AGTATGGAAAGTGTGGCGCT
*CYP3A4*-R	TCCTCAGCTATAGACGTGGTA
*MCL1*-F	AAACGCGGTCATCGGACCA
*MCL1*-R	GCCGTGGCGGAAAACCTC
*MLH1*-F	TGCAGGGGGATACAACTTAGG
*MLH1*-R	TCCACATCACAATCTTCCTGT
miR-3664-3p-F	TCTCAGGAGTAAAGACGGTCAT
Universal reverse primer	ACATTCAGAGTGACTC
miR-3664-3p stem-loop	TAATTAGAGTACATCTGTTGGTACTGTGTCCCTGCGTCTTG CAG TGT TAT GG TTCTC

TaqMan master mix (Ampliqon, Denmark) was used for miRNA expression analysis. To prepare the reaction, 0.4 μM of universal TaqMan probe, 2 μl of cDNA, and 5 pmol of each primer were mixed in a final volume of 20 μl. The thermal cycling protocol consisted of 50 cycles of denaturation (95 °C for 30 s), annealing (58 °C for 30 s), and extension (72 °C for 30 s), following initial heating at 95 °C for 15 min. To normalize gene expression levels, the *U47* transcript was used as the reference gene. Each experiment was performed in triplicate, and PCR efficiency for each assay was determined to be 95%–105 % using standard curve analysis.

To determine the expression levels of mRNAs or miRNAs, relative expression and fold change were calculated based on the mean CT of the replicates. ΔCT values were obtained by comparing the CT of the target gene to that of the housekeeping gene (*U47* for miRNA and *Gapdh* for mRNAs). ΔΔCT values were then computed by subtracting the ΔCT of the untreated samples from that of the treated ones. With a PCR efficiency of 95–105 %, the 2−ΔΔCT formula was applied to determine the fold change of expression, raising 2 to the power of -ΔΔCT.

### 2.6 Pre-miR-3664-3ptransfection in SW480 and HCT116 cell lines by lipofectamine

For transfection, cells were seeded in 6-well plates at a density of 8 × 10^5^ cells per well and incubated for 24 h. The following day, the media was refreshed with new, supplemented media. With 10 % FBS to achieve 80 % confluency. Two days before transfection, the media was switched to antibiotic-free media. For the transfection, 5 μg of the plasmid pLK.1-EGFP-puro (GENERAY, China) were mixed with Lipofectamine 3000 (Invitrogen, Thermo Fisher Scientific, Waltham, MA, USA) at a 1:3 ratio according to the manufacturer's instructions and added to the culture media. After 6 h, the media was refreshed with new, supplemented media containing 10 % FBS. The plate was then incubated at 37 °C with 5 % CO2 for 18 h. To determine transfection efficiency, GFP-expressing cells were counted 24 h post-transfection, resulting in transfection efficiency of 60–70 % for both cell lines.

### 2.7 Statistical methods

The results from three experiments were presented as the mean value along with the standard deviation (SD) with Graph Pad Prism version 8.0.2 (GraphPad Software, La Jol-la, CA, USA). To assess differences between sub-groups, analysis of variance (ANOVA) was performed, followed by the Tukey post hoc test for further interpretation Pearson correlation analysis was used to assess correlations. A p-values less than 0.05 was considered statistically significant.

## 3 Results

### 3.1 Prediction of MicroRNAs targeting *ABCG2*, *CYP3A4*, *MCL1*, and *MLH1* genes

The characteristics of miRNAs targeting the 3′ UTR regions of the *ABCG2, CYP3A4, MCL-1,* and *MLH1* genes were analyzed using a bioinformatics approach. Based on the aforementioned criteria, miRNAs that could simultaneously target *ABCG2, CYP3A4, MCL-1,* and *MLH1* were evaluated, and miR-3664-3p was identified as the most suitable candidate.

**Fig. 1:**
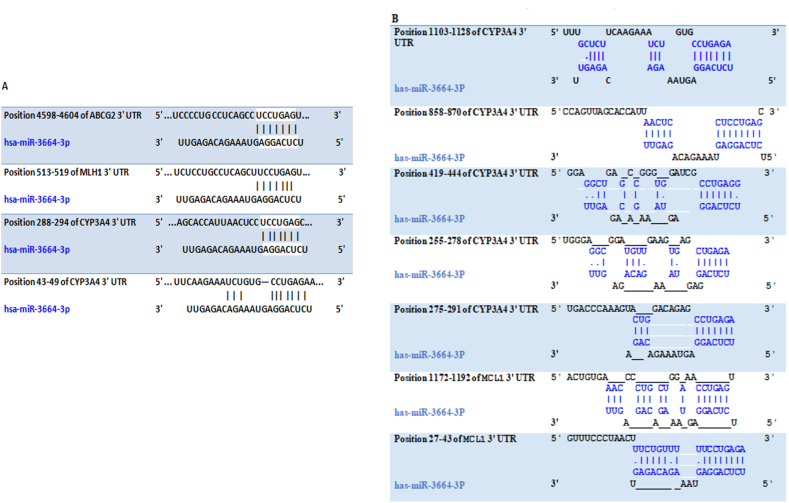
Complementary sequence for miR-3664-3p in 3′UTR of *ABCG2, CYP3A4, MCL1,* and *MLH1* genes. A: targetscan B: Diana tools.

As shown in (Fig. 1A and B), miR-3664-3p exhibited several complementary sequences within the 3′ UTR regions of the *ABCG2, CYP3A4, MCL1,* and *MLH1* genes. Specifically, miR-3664-3p contained one complementary site on the 3′ UTR of the *ABCG2* gene, located at nucleotide positions 4598–4904 nucleotides. Additionally, it had seven complementary sites on the 3′ UTR of the *CYP3A4* gene, found at the following positions 288–294, 43–49, 1103–1128, 858–870, 419–444, 255–278, and 275–291 nucleotides. Furthermore, miR-3664-3p had one attachment site on the 3′ UTR of the *MLH1* gene, located at positions 513–519 nucleotides. Lastly, it had two attachment sites on the 3′ UTR of the *MCL1* gene, located at positions 1172–1192 and 27–43 nucleotides.

### 3.2 After four rounds of developing resistance, HCT116 and SW480 cell lines acquired resistance to irinotecan

**Fig. 2:**
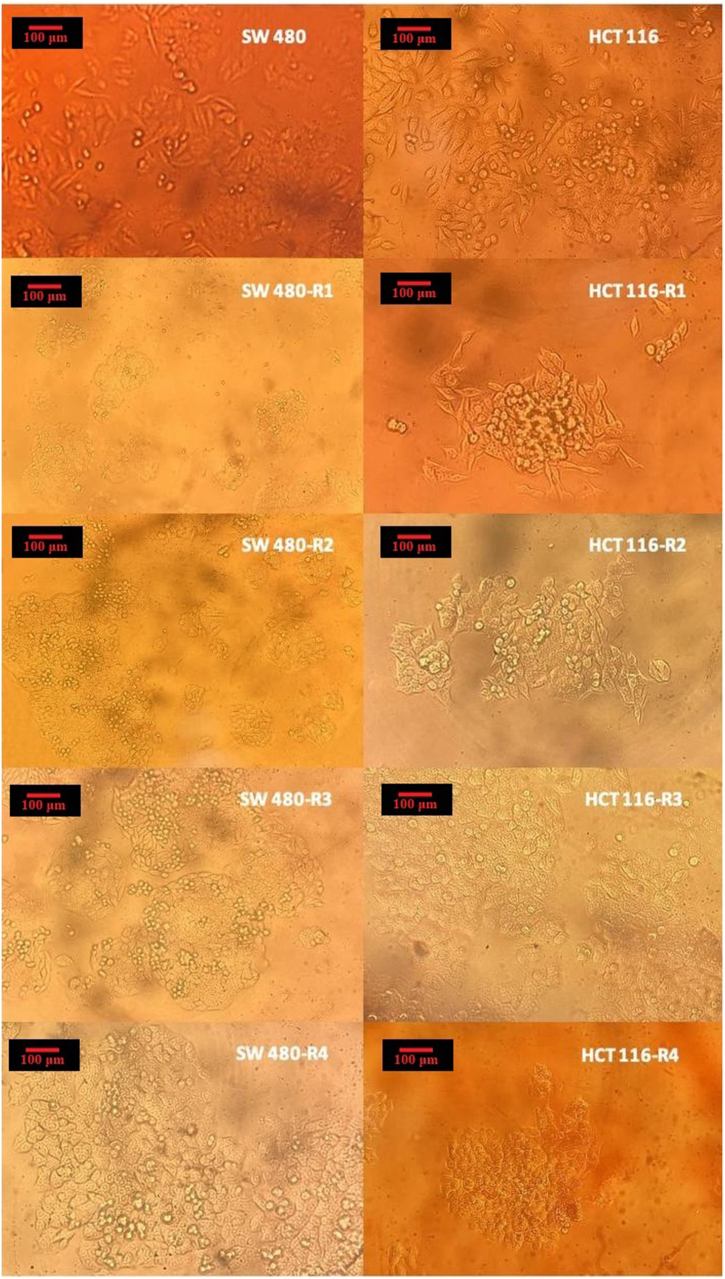
The cell morphology of the parental HCT116 and SW480 cell lines, as well as the irinotecan-resistant cell lines. These images were captured after 1–2 passages of cells, and it is crucial to note that the cells were not treated with irinotecan at the time of imaging. These images provide insight into the morphological changes associated with resistance induction. The pictures were taken from cells under an inverted microscope equipped with a 20X objective lens (magnification × 400; Nikon; Kurobane Nikon Co., Ltd, Otawara, Japan).

**Fig. 3:**
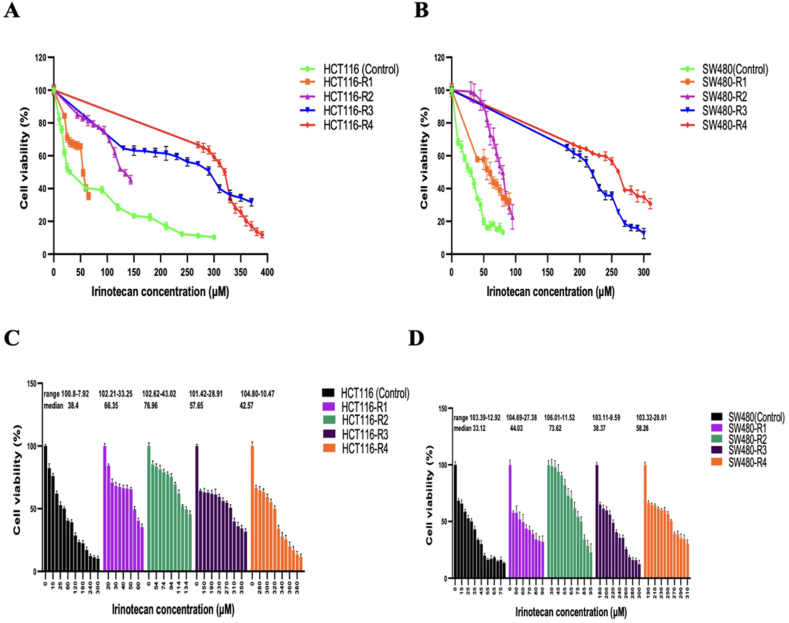
Cell viability in various irinotecan-resistant sub-lines derived from HCT116 and SW480. (A) Cell viability in HCT116-derived sub-lines compared to the parental HCT116 line. The data points represent the mean ± SD. (B) Cell viability in SW480-derived sub-lines compared to the parental SW480 line. The data points represent the mean ± SD. (C) Cell viability in HCT116-derived sub-lines compared to the parental HCT116 line. The lines represent the maximum, median and minimum values. (D) Cell viability in SW480-derived sub-lines compared to the parental SW480 line. The lines represent the maximum, median and minimum values. Figures were generated using GraphPad Prism.

The resistant cell lines were created sequentially through treatments with irinotecan. The IC_50_ of the parental HCT116 cell line was measured at 30 ± 1.49 μM. In comparison, the IC_50_ values for the resistant cell lines for HCT116-R1, HCT116-R2, HCT116-R3, and HCT116-R4 were 55 ± 2.06 ng/μl, 134 ± 2.03 ng/μl, 290 ± 2.45 ng/μl, and 320 ± 1.97 ng/μl, respectively. These IC_50_ values showed a significant increase from the parental HCT116 cell line, with HCT116-R4 demonstrating an increase of approximately 10.66-fold (Fig. 3A). The range and median of cell viability for several cell lines are illustrated in Fig. 3C. Morphological observations indicated that as the resistant cell lines became increasingly resistant with each induction round, their phenotypic characteristics also changed. The cells exhibited reduced size and adherence, while cell-cell interactions increased, leading to more clustered formations. This clustering phenomenon may provide a survival advantage by offering protection against the cytotoxic effects of chemotherapy drugs (Fig. 2).

Four generations of SW480-resistant cell lines were developed through consecutive treatments with irinotecan. The IC_50_ of the parental SW480 cell line was 30 ± 2.64 ng/μl. In comparison, the IC_50_s of SW480-R1, SW480-R2, SW480-R3, and SW480-R4 were 60 ± 6.57 ng/μl, 80 ± 4.08 ng/μl, 220 ± 2.46 ng/μl, and 260 ± 1.11 ng/μl, respectively. These values indicate a significant increase in resistance compared to the parental cell line. Notably, the IC_50_ of SW480-R4 increased approximately 8.66 times relative to the parental SW480 cells (Fig. 3B). Additionally, the range and median of cell viability for various cell lines are displayed in Fig. 3D. After developing resistance, the SW480-R4 showed a decrease in cell size and exhibited characteristics of loosely attached cells, often forming sparsely populated clusters. Interestingly, these clusters displayed a circular arrangement with equal distances between them, suggesting a high level of organization and coordination among the cells (Fig. 2).

### 3.3 The expressions of *ABCG2* in all HCT116 andSW480-derived resistant cell lines showed a significant increase compared to the parental cell line

**Fig. 4:**
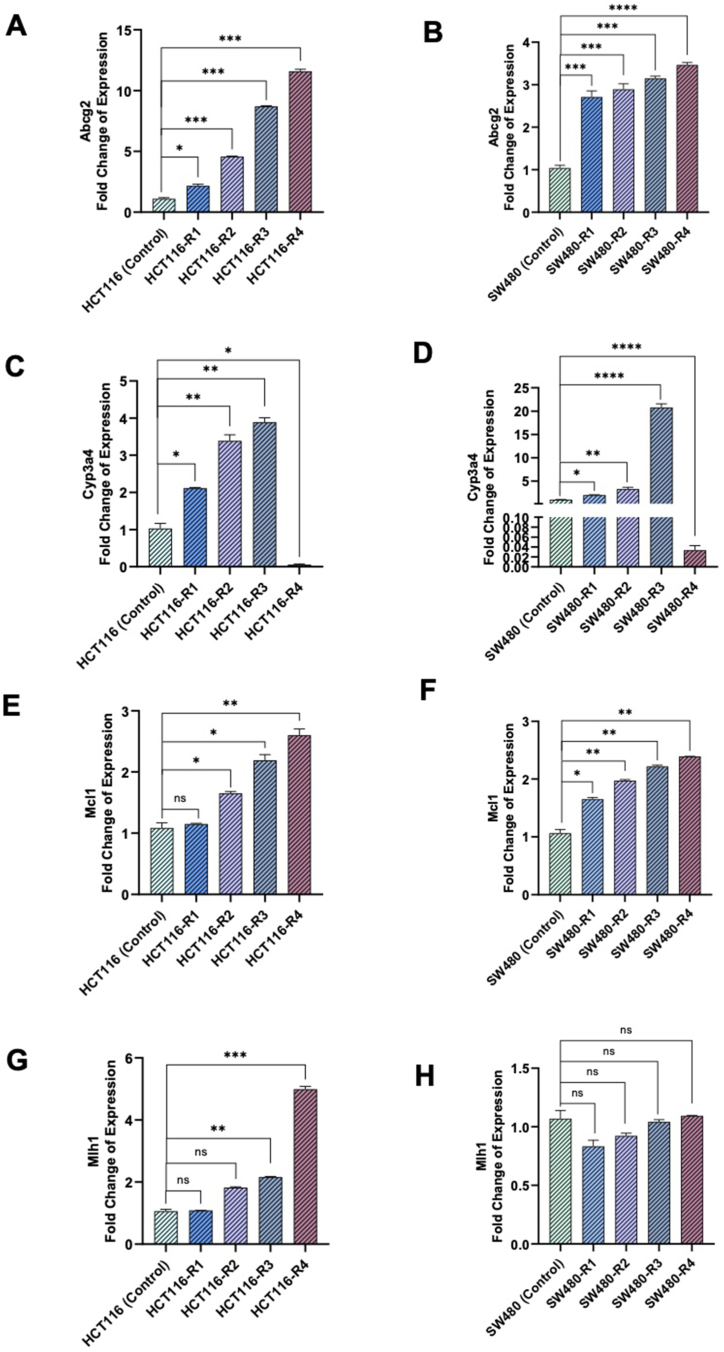
Real-time PCR quantification of *ABCG2* transcript levels in (A) HCT116 and (B) SW480 parental and derived cell lines. *CYP3A4* transcript levels in(C) HCT116 and (D) SW480 in parental and derived cell lines. *MCL1* transcript levels in (E) HCT116 and (F) SW480 parental and derived cell lines. *MLH1* transcript levels in (G) HCT116 and (H) SW480 parental and derived cell lines. The data are presented as the Mean ± Std. Error of Mean (SEM). ∗ denotes p value < 0.05, ∗∗ denotes p value < 0.005, ∗∗∗ stands p value < 0.0005, and ∗∗∗∗ stands p value < 0.0001. ns: not significant. Statistical analysis was conducted using Ordinary One-way ANOVA. Graphs were created using GraphPad Prism version 8.0.2.

The fold changes in *ABCG2* expression in the HCT116-R1, HCT116-R2, HCT116-R3, and HCT116-R4 cell lines, compared to the parental HCT116 cell line, were 2.16 ± 0.17, 4.57 ± 0.10, 8.71 ± 0.11, and 11.59 ± 0.19, respectively. These differences were statistically significant (Fig. 4A). Similarly, the fold changes in *ABCG2* expression in the SW480-R1, SW480-R2, SW480-R3, and SW480-R4 cell lines were 2.71 ± 0.15, 2.89 ± 0.12, 3.14 ± 0.09, and 3.46 ± 0.08, respectively, all of which were also significantly different from the parental cell line (Fig. 4B).

### 3.4 Elevated levels of *CYP3A4* expression were observed in the first three resistant cell lines of HCT116 and SW480 while the level of this gene was reduced in HCT116-R4 and SW480-R4

When compared to the parental HCT116 cell line, the fold changes in *CYP3A4* expression in HCT116-R1, HCT116-R2, and HCT116-R3 were 2.11 ± 0.21, 3.38 ± 0.34, and 3.89 ± 0.47, respectively (Fig. 4C). Similarly, for SW480-R1, SW480-R2, and SW480-R3, the fold changes in expression were 2.0 ± 0.07, 3.27 ± 0.20, and 20.78 ± 0.57, respectively. These values were notably distinct from the expression levels in the parental SW480 cell line (Fig. 4D). Surprisingly, HCT116-R4 and SW480-R4, exhibited CYP3A4 expression levels of 0.05 ± 0.14 and 0.03 ± 0.04, respectively, compared to the parental cell lines. These levels represent a significant decrease compared to the expression in HCT116-R3 and SW480-R3 cell lines (Fig. 4C and D).

### 3.5 Elevated expression of the *MCL1* gene compared to the parental cell lines was observed in HCT116-R3, HCT116-R4, and SW480-R3, SW480-R4

In HCT116-R3 and HCT116-R4, the fold changes in *MCL1* expression, compared to the parental HCT116 cell line, were 2.19 ± 0.3 and 2.60 ± 0.21, respectively (Fig. 4E). Similarly, in SW480-R3 and SW480-R4, the fold changes in *MCL1* expression were 2.22 ± 0.068 and 2.39 ± 0.065, respectively, both showing significant differences compared to the parental SW480 cell line (Fig. 4F). In HCT116-R1 and HCT116-R2, the fold changes were 1.15 ± 0.084 and 1.65 ± 0.089, respectively (Fig. 4E). Similarly, in SW480-R1 and SW480-R2, the fold changes were 1.65 ± 0.071 and 1.97 ± 0.066, respectively, but did not reach statistical significance (Fig. 4F).

### 3.6 *MLH1* expression was upregulated in HCT116-R3 and HCT116-R4 compared to the parental cell lines while no changes in MLH1 expression were observed in any of the SW480-derived resistant cell lines

The fold changes in *MLH1* expression in HCT116-R3 and HCT116-R4 were 2.16 ± 0.06 and 4.99 ± 0.10, respectively, indicating a notable increase compared to the parental HCT116 cells. In contrast, the fold changes in *MLH1* expression in HCT116-R1 and HCT116-R2 were 1.08 ± 0.06 and 1.82 ± 0.4, respectively, which were below the significant threshold of a 2-fold change (Fig. 4G).

For the SW480-derived resistant cell lines, all observed fold changes in *MLH1* expression were below the 2-fold threshold. Specifically, the expression fold changes were 0.83 ± 0.087 for SW480-R1, 0.92 ± 0.073 for SW480-R2, 1.04 ± 0.072 for SW480-R3, and 1.09 ± 0.07 for SW480-R4 (Fig. 4H).

### 3.7 The expression of miR-3664-3p was reduced following resistance induction in CRC cell lines

**Fig. 5:**
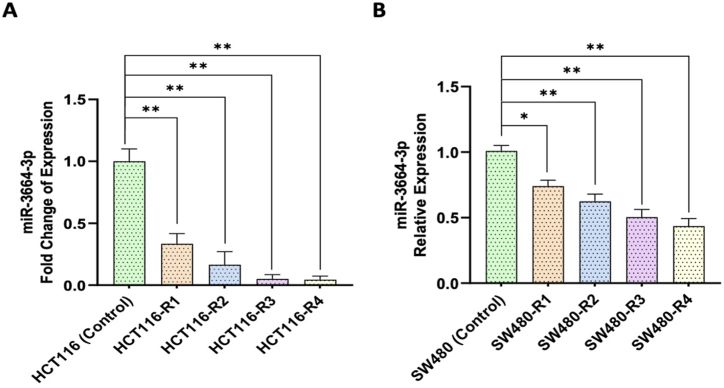
Real-time PCR quantification of miR-3664-3p: (A) Resistant cell lines derived from HCT116, and (B) resistant cell lines derived from SW480. The data are expressed as the mean ± Std. Error of Mean (SEM). ∗ denotes p value < 0.05, ∗∗ denotes p value < 0.005, ∗∗∗ denotes p value < 0.0005, and ∗∗∗∗ denotes p value < 0.0001. ns: not significant. Statistical evaluation was performed using Ordinary One-way ANOVA. Figures were generated using GraphPad Prism version 8.0.2.

Fig. 5A illustrates that the levels of miR-3664-3p decreased progressively in all resistant cell lines derived from HCT116. The reduction observed were as follows: 0.33 ± 0.06 for HCT116-R1, 0.16 ± 0.07 for HCT116-R2, 0.05 ± 0.08 for HCT116-R3, and 0.04 ± 0.07 for HCT116-R4. Similarly, a reduction in miR-3664-3p expression was noted in SW480-R3 and SW480-R4, with values of 0.50 ± 0.07 and 0.43 ± 0.07, respectively, compared to the parental SW480 cell line. However, in SW480-R1 and SW480-R2, the decrease was not statistically significant, with values of 0.74 ± 0.08 and 0.62 ± 0.06, respectively (Fig. 5B).

### 3.8 The expression level of miR-3664-3p exhibited an inverse correlation with *ABCG2, CYP3A4, MCL1*, and *MLH1* levels in all resistant cell lines

**Fig. 6:**
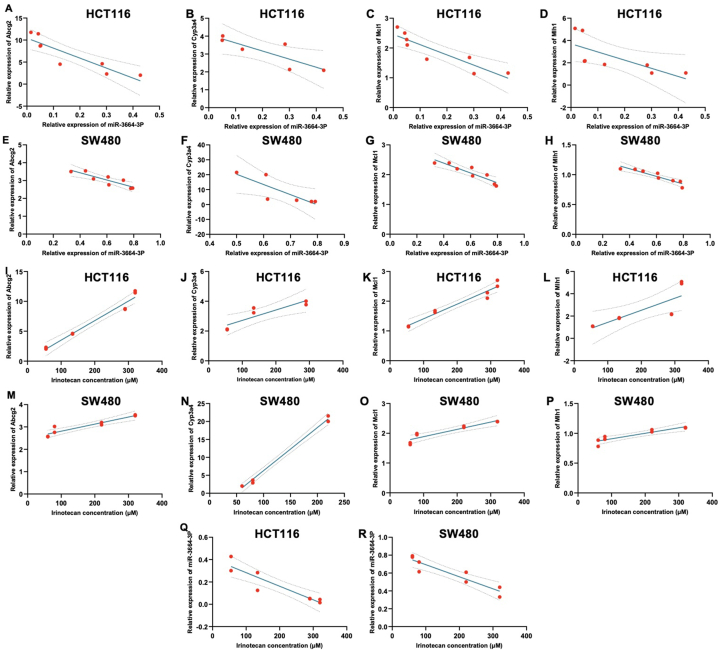
Correlation between miR-3664-3p level and *ABCG2* transcript level in(A) HCT116 and(B) SW480-derived cell lines, *CYP3A4* transcript level in (C) HCT116-R1, HCT116-R2, and HCT116-R3 and (D)SW480-R1, SW480-R2, and SW480-R3 cell lines, *MCL1* transcript level in-(E) HCT116, and (F) SW480-derived cell lines. *MLH1* transcript level in all (G) HCT116 and (H) SW480-derived cell lines. Correlation between IC_50_ to irinotecan and *ABCG2* transcript level in all (I) HCT116 and (J) SW480-derived cell lines. *CYP3A4* transcript level in (K) HCT116-R1, HCT116-R2, and HCT116-R3, and (L) SW480-R1, SW480-R2, and SW480-R3. *MCL1* transcript level in all (M) HCT116 and (N) SW480-derived cell lines *MLH1* transcript level in all (O) HCT116 and (P) SW480-derived cell lines. Correlation between the sensitivity of cells to different irinotecan concentrations and miR-3664-3p level in all (Q) HCT116, and(R) SW480-derived cell lines. Curves were generated using GraphPad, illustrating the correlation between the expression of genes and miRNA and the irinotecan IC_50_. Each dot represents the status of individual cells based on their sensitivity to irinotecan and the *ABCG2*, *CYP3A4, MCL1*, *MLH1* genes, and miR-3664-3p expression levels. This analysis was conducted using data from three replicates, and the figures were generated with GraphPad Prism.

The Pearson correlation test demonstrated a strong inverse correlation between miR-3664-3p levels and the transcript levels of *ABCG2, CYP3A4, MCL1,* and *MLH1* across all resistant cell lines derived from SW480 and HCT116. Notably, miR-3664-3p levels were inversely correlated with *ABCG2* levels in cell lines HCT116-R1, HCT116-R2, HCT116-R3, HCT116-R4, as well as SW480-R1, SW480-R2, SW480-R3, and SW480-R4. The correlation coefficients of r = −0.8983 (P = 0.0024) for the cell lines derived from HCT116, and r = −0.8815 (P = 0.0038) for those derived from SW480 (Fig. 6A and B).

MiR-3664-3p levels were found to be inversely correlated with *CYP3A4* levels in HCT116-R1, HCT116-R2, HCT116-R3, and SW480-R1, SW480-R2, and SW480-R3. The correlation values were r = −0.8413 (P = 0.0358) for HCT116-derived cell lines and r = −0.8287 (P = 0.0415) for SW480-derived cell lines, respectively (Fig. 6C and D).

Moreover, miR-3664-3p levels negatively correlated with expression levels of *MCL1* and *MLH1* in all cell lines derived from SW480 and HCT116. Specifically, the correlation coefficients between miR-3664-3p levels and *MCL1* expression were r = −0.8988 (P = 0.0024) for HCT116-derived cell lines and r = −0.9141 (P = 0.0015) (Fig. 6E and F). For MLH1 expression, the correlation coefficients and SW480-derived cell lines were r = −0.7149 (P = 0.0463) for HCT116-derived cell lines and r = −0.9310 (P = 0.0008) for SW480-derived cell lines (Fig. 6G and H).

The findings indicate that as miR-3664-3p levels decreased in the resistant cell lines, the *ABCG2*, *CYP3A4*, *MCL1*, and *MLH1* expression levels increased. This suggests that miR-3664-3p may play a regulatory role in the expression of these genes during the process of developing resistance to irinotecan. Overall, the evidence strongly indicates that miR-3664-3p regulates the expression of these four genes.

### 3.9 The IC_50_ of irinotecan showed a positive correlation with the expression levels of *ABCG2, CYP3A4, MCL1,* and *MLH1* in all cell lines derived from HCT116 and SW480

The Pearson correlation test confirmed the positive correlation between the IC_50_ values of irinotecan and the expression levels of *ABCG2, CYP3A4, MCL1,* and *MLH1* across all SW480- and HCT116-derived cell lines. Specifically, the correlation coefficient between IC_50_ and the expression of *ABCG2* in all HCT116 and SW480 cell lines was r = 0.9821 (P = 0.0001) and r = 0.9432 (P = 0.0004), respectively (Fig. 6I and J). Similarly, for the expression of *CYP3A4* expression in HCT116-R1, HCT116-R2, HCT116-R3, and SW480-R1, SW480-R2, and SW480-R3, the correlation coefficient were r = 0.8968 (P = 0.0154) and r = 0.9968 (P = 0.0001), respectively (Fig. 6K and L). Moreover, the correlation coefficients between IC_50_ and the expression levels of *MCL1* and *MLH1* across all HCT116-and SW480-derived cell lines were r = 0.9743 (P = 0.0001), r = 0.9321 (P = 0.0007), for *MCL1*, and r = 0.7990 (P = 0.0174), and r = 0.9187 (P = 0.0013) for *MLH1* respectively (Fig. 6M, N, 6O, 6P). These findings suggest that, as the resistance to irinotecan developed, the levels of *ABCG2, CYP3A4, MCL1,* and *MLH1* increased as the cells developed resistance to irinotecan.

### 3.10 The IC_50_ of irinotecan was inversely correlated with the miR-3664-3p level in all HCT116 and SW480-derived cell lines

Pearson correlation analysis was conducted to examine the relationship between cell sensitivity to various concentrations of irinotecan and the level of miR-3664-3p. The analysis demonstrated a strong inverse correlation between miR-3664-3p levels and IC_50_ in all HCT116 (r = −0.9198, p = 0.0012) and SW480-derived (r = −0.9261, p = 0.0010) cell lines (Fig. 6Q and R). These findings suggested that as resistance to irinotecan was induced, there was a consistent decrease in the level of miR-3664-3p.

### 3.11 miR-3664-3p enhanced the sensitivity of SW480 and HCT116 cell lines to irinotecan

**Fig. 7:**
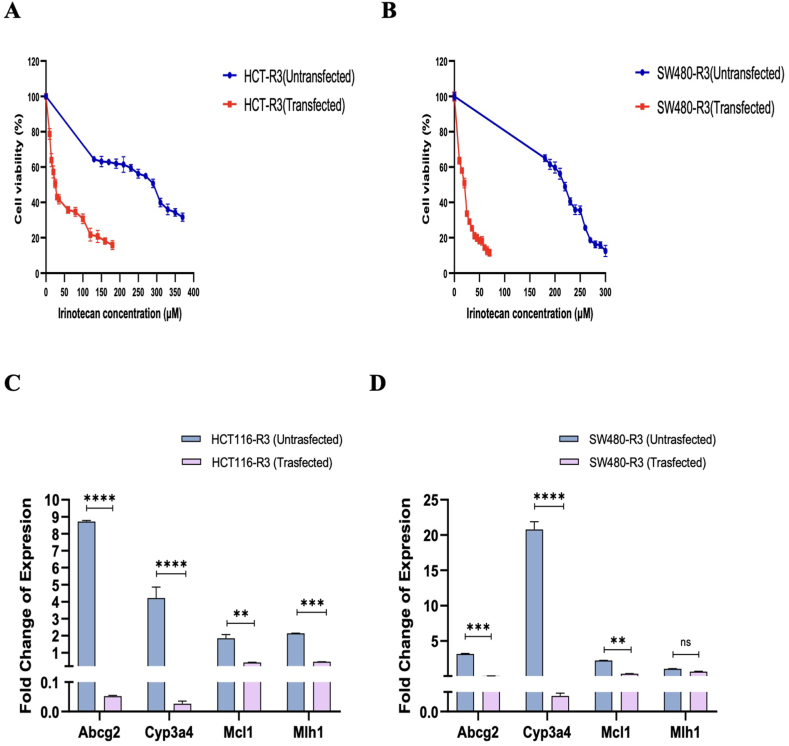
The cell viability assay was performed on transfected (A) HCT116-R3 and (B) SW480-R3 cells, as well as non-transfected cells, using various concentrations of irinotecan (μM). Real-time PCR quantification was conducted to measure the expression levels of *ABCG2, CYP3A4, MCL1, MLH1*, and miR-3664-3p in (C) HCT116-R3 and (D) SW480-R3 cell lines. The data are expressed as the mean ± Std. Error of Mean (SEM). ∗ denotes a p value < 0.05, ∗∗ denotes a p value < 0.005, ∗∗∗ denotes a p-value<0.0005, and ∗∗∗∗ denotes a p value < 0.0001. “ns” stands not significant. Statistical analysis was conducted using Ordinary One-way ANOVA.

To investigate whether miR-3664-3p affected the sensitivity of HCT116-R3 and SW480-R3 cells to irinotecan, these cell lines were transfected with a plasmid containing pre-miR-3664-3p. Following transfection, an MTT assay was performed, exposing the transfected cells to various concentrations of irinotecan. The findings showed that the IC_50_ values of both HCT116-R3 and SW480-R3 cells decreased compared to their parental IC_50_ values. Specifically, the IC_50_ of HCT116 cells decreased from 30 ± 1.49 ng/μl to approximately 25 ± 2.37 ng/μl, while the IC_50_ of SW480 cells decreased from 30 ± 2.64 ng/μl to about 20 ± 2.72 ng/μl in the transfected cells. These findings suggested that transfection with miR-3664-3p increased the sensitivity of cells to irinotecan (Fig. 7A and B). This decrease in IC_50_ reflects an enhanced sensitivity to irinotecan in both pre-miR-3664-3p-transfected cell lines.

### 3.12 Following transfection of HCT116-R3 and SW480-R3 with a plasmid containing pre-miR-3664-3p, the expression levels of *ABCG2, CYP3A4, MCL1*, and *MLH1* decreased

To investigate the effect of miR-3664-3p on the transcript levels of *ABCG2, CYP3A4, MCL1,* and *MLH1* genes, pre-miR-3664-3p was transfected into both HCT116-R3 and SW480-R3 cell lines. Post-transfection, there was a substantial increase in miR-3664-3p expression, with the levels in HCT116-R3 and SW480-R3 cell lines being 75.27 and 81.83 times higher, respectively, than in non-transfected cells.

In the transfected HCT116-R3 cells, the expression level of the *ABCG2* was measured at 0.05 ± 0.04, while in the transfected SW480-R3 cells, it was 0.03 ± 0.04. These results indicate a notable decrease in the *ABCG2* expression in both cell lines. Likewise, the expression level of the *CYP3A4* gene decreased in the transfected HCT116-R3 cells recorded at 0.03 ± 0.44. In the SW480-R3 cell line, this gene's expression was reduced even further, to 0.0008 ± 0.44.

Additionally, the expression level of the *MCL1* gene decreased in both HCT116-R3 cells (0.42 ± 0.16) and SW480-R3 cells (0.34 ± 0.16) following transfection. However, while the *MLH1* gene expression level decreased significantly in transfected HCT116-R3 cells (0.46 ± 0.007), it did not decrease significantly in transfected SW480-R3 cells (0.64 ± 0.007), as it remained above the 0.5-fold change threshold (Fig. 7C and D). Conclusively, after transfection with miR-3664-3p expressing plasmid, the expression levels of *ABCG2*, *CYP3A4*, and *MCL1* decreased in both cell lines while the *MLH1* level only decreased in SW480-R3.

## 4 Discussion

Patients with colorectal cancer (CRC) often face the risk of local recurrence or metastasis after surgery, particularly when diagnosed at an advanced stage where complete surgical resection is no longer feasible. These challenges underscore the critical role of chemotherapy in the treatment of CRC. Currently, irinotecan (CPT-11), used either alone or in combination with other agents, helps alleviate symptoms in patients with advanced CRC. However, the 5-year survival rate for those with metastatic or recurrent CRC remains below 10 %, illustrating the challenges associated with chemotherapy resistance. The development of this resistance significantly hinders the achievement of satisfactory therapeutic outcomes with CPT-11 treatment. To improve the success rate of chemotherapeutic regimens, it is essential to have a comprehensive understanding of the mechanisms underlying chemotherapy resistance to identify potential agents to reverse it [7]. Consequently, further research into the mechanisms that induce drug resistance is urgently needed. One promising strategy to overcome chemotherapy resistance in CRC is the use of microRNAs (miRNAs) [38].

The ability of selectively chosen miRNAs to target multiple mRNAs makes them promising candidates for modulating disease conditions. This therapeutic strategy can be applied through miRNA mimics, microRNA-regulated vectors, or anti-miRs, both in vivo and in vitro. Additionally, advancements in RNA delivery technologies have made miRNA-based therapeutics a feasible option [39]. These small molecules are associated with various chemo-resistant mechanisms, and targeting or utilizing them has shown promising results in addressing CRC chemo-resistance [38]. Cancer cells can develop resistance to irinotecan through intrinsic or acquired mechanisms, involving various genes and molecular pathways [[32], [34], [40]]. The overexpression of ATP-binding cassette (ABC) transporters, including *Abcb1, ABCG2*, and *Abcc1*, is essential in facilitating drug efflux and conferring resistance. Specifically, overexpression of *ABCG2* in several cancer cell lines, such as breast cancer, colon carcinoma, and stomach cancer, contributes to resistance to various anticancer drugs. This highlights its role as a multi-drug resistance (MDR) factor, which is related to both intrinsic and acquired chemo-resistance [[35], [36], [37]].

Suppressing ABCG2 function using Ko143 resulted in increased drug accumulation and enhanced sensitivity to the drug in resistant cells, underscoring the pivotal function of ABCG2 in acquired resistance [41]. Recent findings by Nielsen et al. emphasize the significance of *ABCG2* upregulation in acquired resistance to SN-38 in both breast cancer and CRC cell lines [42].

CYP3A4 plays a crucial role in innate and acquired resistance to irinotecan by metabolizing the drug into active and inactive forms, impacting its efficiency and toxicity [13]. Genetic variations in *CYP3A4* can lead to alterations in enzyme activity, which may influence irinotecan metabolism and contribute to intrinsic chemo-resistance [13]. In the case of acquired resistance, the steroid and xenobiotic receptor (SXR) and retinoid X receptor (RXR) are critical in regulating *CYP3A4* expression. Upon exposure to irinotecan, SXR becomes activated moves to the nucleus, and forms a heterodimer with RXR. This SXR-RXR complex then binds to the *CYP3A4* promoter region, inducing *CYP3A4* expression. The subsequent increase in CYP3A4 levels facilitates irinotecan detoxification, thus promoting acquired resistance in cancer cells [43]. Rodríguez et al. found that peripheral T-cell lymphomas (PTCLs), which are known for their aggressive nature, consistently exhibit high levels of CYP3A4. This elevated expression is significantly associated with a reduced rate of complete remission and heightened resistance to chemotherapeutic agents doxorubicin and etoposide [44].

MCL1, an anti-apoptotic protein within the BCL-2 family, is frequently overexpressed in various cancers, including colon, esophageal, and ovarian cancers, as well as multiple myeloma, leukemia, *and* lymphoma [[37], [45], [46], [47], [48], [49]]. This overexpression contributes to chemo-resistance by enabling cancer cells to evade programmed cell death and senescence, which are induced by chemotherapy drugs like irinotecan. Jonchére et al. demonstrated that an irinotecan-resistant HCT116 cell line exhibited increased malignancy. These cells formed tumors more readily when injected into mice and displayed reduced cell adhesion. The malignant cells also exhibited resistance to senescence through MCL1 and BCL-X1 signaling pathways. Deletion of *MCL1* improved irinotecan efficacy, induced cell death in polyploid cells, and inhibited the growth of less adherent cells [50]. Feng et al. found that USP20 regulates the de-ubiquitination of MCL1, thereby affecting its stability. They noticed that elevated USP20 expression is associated with higher levels of MCL1 protein in esophageal cancer cell lines. Depletion of USP20 resulted in increased poly-ubiquitination of MCL1, which in turn enhanced MCL1 turnover and increased the sensitivity of cells to chemotherapy [51].

*MLH1,* a gene responsible for DNA mismatch repair*,* has a controversial role in chemo-resistance. Increased MLH1 expression has been linked to resistance to certain drugs, while decreased expression is also associated with resistance to others [[20], [52]]. For example, increased *MLH1* expression can contribute to chemotherapy resistance to drugs like irinotecan by enhancing DNA repair, thereby reducing the drug's cytotoxic effects. As a result, cancer cells with elevated *MLH1* levels may evade irinotecan-induced cytotoxicity, allowing for continued proliferation [53].

Bendardaf et al. demonstrated that mismatch repair status serves as a predictive indicator of tumor response to irinotecan and 5-FU in patients with advanced-stage colorectal cancer. Patients with MMR defects tend to metastasize earlier and are more likely to respond favorably to 5-FU combined with folic acid chemotherapy [54]. Similarly, Tentori et al. showed that MLH1, in combination with reduced levels of TOP1, contributes to colon cancer's resistance to irinotecan. *MLH1*-proficient cells exhibit lower DNA damage caused by SN-38 compared to *MLH1*-deficient cells. In the *MLH1*-deficient HCT-116 cell line, treatment with SN-38 triggered a dose-dependent elevation in p53 phosphorylation and apoptosis, effects that were more pronounced in PARP-1-silenced cells [55]. Manzoor et al. found that 5-FU and irinotecan did not significantly affect the *MLH1*-proficient HCT116 and SW480 cell lines [20].

The *MLH1* gene is crucial for the DNA mismatch repair (MMR) mechanism. When this gene loses its activity, it can lead to chemoresistance, particularly for drugs such as cisplatin. MLH1 often collaborates with other proteins to rectify DNA replication errors. If MLH1 becomes dysfunctional, these errors can accumulate, making the cells more resistant to chemotherapy-induced DNA damage [56]. According to research by Zeller et al. ovarian cancer patients who undergo platinum-based chemotherapy may develop hypermethylation of the *MLH1* gene, resulting in gene inactivation, and poorer clinical outcomes. Studies have shown that re-expressing *MLH1* in chemo-resistant cell lines can partially restore their sensitivity to cisplatin therapy [57].

Activation of the PI3K/AKT pathway contributes to multidrug resistance (via ABCG2), alters the drug metabolism (via CYP3A4), and enhances cell survival (via MCL1), all of which play key roles in cancer cell resistance to chemotherapy drugs like irinotecan [[21], [22], [23]]. Inhibition of PI3K/AKT signaling pathway by agents like LY294002 or rapamycin has been shown to reduce ABCG2 expression in human multiple myeloma (MM) cell lines, suggesting that PI3K/AKT signaling positively regulates ABCG2 levels [21]. The PI3K/AKT pathway also activates CYP3A4, primarily through its effects on FOXO1, enhancing the transcriptional activity of the pregnane X receptor (PXR). PXR, by binding to the *CYP3A4* promoter region, ultimately increases CYP3A4 level [22]. The PI3K/AKT signaling pathway also influences MCL1 expression. Upon AKT phosphorylation, cell survival is promoted by inactivating pro-apoptotic proteins and regulating anti-apoptotic proteins. When AKT is activated, MCL1, an anti-apoptotic protein, is upregulated through increased expression or stabilization [23]. Although no specific pathway has been definitively identified as a direct modulator of *MLH1* expression, DNA repair-related pathways and proteins can influence *MLH1* gene expression. For instance, the p53 protein enhances the activity of mismatch repair (MMR) proteins, including MLH1, thereby playing a crucial role in maintaining genomic stability [24]. MiRNAs targeting different targets within a pathway can effectively modulate these pathways. Similar target sites in 3′UTR of these genes may imply a shared role in certain pathways. Given that the online methods predict that miR-3664-3p simultaneously targets the 3′UTR of *ABCG2*, *CYP3A4*, *MCL1*, and *MLH1* genes, we investigated the expression of these genes and this miRNA in the resistance process to irinotecan. AKT was correlated with the increased expression of *ABCG2*, *CYP3A4*, and *MCL1* along with multidrug resistance. Thus, miR-3664-3p may function as an inhibitor of this pathway by downregulating the downstream genes. Inhibiting this pathway may lead to enhanced sensitivity to irinotecan.

In our study, the induction of resistance to irinotecan in two colon cancer cell lines (HCT116 and SW480) resulted in increased expression of *ABCG2, CYP3A4,* and *MCL1*. At the same time, *MLH1* expression also increased in resistant cells derived from HCT116. Despite elevated *ABCG2*, *CYP3A4*, and *MCL1* levels in resistant cells derived from SW480, *MLH1* expression showed only a slight increase. In all resistant cell lines, except for HCT116-R4 and SW480-R4, the *CYP3A4* mRNA level was correlated with the IC_50_. The reduction in *CYP3A4* gene expression in the HCT116-R4 and SW480-R4 cell lines may be attributed to the activation of specific molecular signaling pathways, such as the PXR-mediated negative feedback loop [58]. On the other hand, abnormal DNA methylation patterns are commonly observed in cancer and can significantly impact the expression of drug-metabolizing enzymes like CYP3A4. Increased methylation in the promoter regions of *CYP3A4* may result in reduced enzyme expression, particularly in tumor cells [59]. Cancer cells typically rely on anaerobic glycolysis, which is less efficient for ATP production but supports the biomass production necessary for cell proliferation. This metabolic shift suggests that when a cell overexpresses enzymes like CYP3A4, which require substantial resources, it could divert nutrients from pathways critical for growth. Consequently, in cancer cells, the expression of these genes may be reduced to conserve energy for survival [60]. Furthermore, cancer cells function within intricate gene regulatory networks that determine their responses to various treatments. Once drug resistance is established, some genes that were once vital for survival, such as *CYP3A4* (involved in drug metabolism), may become less critical as the cells adapt to the presence of chemotherapy agents. At the same time, other genes may be upregulated to compensate for this loss of function. In this manner, cancer cells can maintain their resilience to therapeutic agents through compensatory gene activity [61].

In summary, although CYP3A4 initially contributes to drug resistance, the subsequent decrease in its expression indicates an adaptive optimization. This means being more dependent on other effective resistance mechanisms. This transition helps cancer cells manage their resources more efficiently, enhancing their survival ability while still resisting chemotherapy drugs [[58], [59], [60], [61]].

Previous studies have shown variable expression patterns in different resistant cell lines. For example, during the first three stages of developing resistance to oxaliplatin, the cell lines did not show an increase in *Abcb1* expression. However, in the HCT116 cell line, which demonstrated the highest level of resistance, the expression of this gene was elevated. Conversely, an increase in *Abcb1* expression was noticed in the third round of resistance induction of the SW480-derived cell line [27]. Furthermore, a study by Rajabpour et al. evaluated the expression levels of the *Rrm1* and *Cda* genes during resistance induction to gemcitabine in AsPC-1 and MIA PaCa cell lines. The increased expression of these two genes began from the 2nd round of resistance induction [32].

In our study, we observed that the expression of *ABCG2* and *CYP3A4* increased from the first round of resistance induction. Additionally, in the third round of resistance induction, elevated expression of *MCL1* and *MLH1* was detected. This finding suggests that, during the induction of chemo-resistance to irinotecan, the *ABCG2,* and *CYP3A4* genes exhibit earlier and more pronounced responses compared to *MLH1* and *MCL1*.

In a study by Candeil et al., a sensitive HCT-116 clone was subjected to increasing SN38 doses. This led to the isolation of two resistant clones: HCT116-SN6 and HCT116-SN50. These clones demonstrated 6.1- and 53.2-fold resistance to SN38, respectively, when compared to the sensitive clone. This resistance was linked to the increased expression of the *ABCG2* gene [41]. In another study by Tang et al., drug-resistant cell lines H460/CisR and A549/CisR were developed through repeated exposure to cisplatin. A significant increase in *ABCG2* expression was observed in these lines. The IC_50_ value for the cisplatin-resistant cells was 7.28 and 7 times higher than that of the original parental cell line for both H460/CisR and A549/CisR cells, respectively [62]. In our study, the IC_50_ of HCT116-R4 cells showed a 10.6-fold increase compared to the parental cell line, which also correlated with an increased level of *ABCG2.*

The 3′ UTRs of *ABCG2, CYP3A4, MCL1,* and *MLH1* genes through miR-3664-3p could result in more effective gene inhibition and increased sensitivity to irinotecan. The *ABCG2, CYP3A4, MCL1*, and *MLH1* genes are overexpressed in various cancers [[36], [61], [62], [63]] and are involved in chemoresistance to multiple drugs[[13], [22], [35], [37], [61], [63]]. Investigating potential off-target effects and other downstream pathways this miRNA influences is crucial for future research. This is particularly true In SW480-derived cells resistant to 5-FU, *ABCG2* expression progressively increased during resistance induction, reaching an 8.72-fold increase by the fifth round. In our study, we observed a 3.46-fold increase in *ABCG2* expression during the fourth round of resistance induction [33].

We transfected the third generation of HCT116 and SW480 cell lines with miR-3664-3p to investigate its effect on the expression of four genes, including *CYP3A4.* After transfecting HCT116-R3 and SW480-R3 with pre-miR-3664-3p, we observed a decrease in the expression of all four genes, which coincided with a reduction in the IC_50_ values for irinotecan. Similarly, Yuling Li et al. noticed that transfecting cisplatin-resistant SiHa/DDP and HeLa/DDP cells with miR-4739 mimics led to reduced *Rhbdd2* mRNA levels and a notable decrease in IC_50_ for cisplatin in both SiHa/DDP and HeLa/DDP cells [64]. After transfection with pre-miR-3664-3p, the IC_50_ of HCT116-R3 and SW480-R3 decreased to 25 and 20, respectively, down from an original IC_50_ of 30 in the parental cell lines. Previous research uncovered that transfecting MIAPaCa-RG4 with pre-miR-608 also reduced the IC_50_ of gemcitabine compared to its parental line, while this same pre-miRNA did not significantly affect the IC_50_ of AsPC-RG2 [32]. Additionally, Wu et al. showed that increased levels of miR-302c restored sensitivity to temozolomide in temozolomide-resistant U251MG-TMZ and LN229-TMZ cells [65]. In agreement with these findings, we observed enhanced sensitivity to irinotecan in HCT116-R3 and SW480-R3 cells following transfection with the pre-miR-3664-3p-expressing plasmid. Through in silico analysis using various online tools, we identified that miR-3664-3p targets the 3′ UTRs of the *ABCG2, CYP3A4, MCL1,* and *MLH1 genes*. In transfected cells, elevated levels of miR-3664-3p corresponded with a reduction in the expression of each of these genes. This suggests that the chemo-sensitizing effect of miR-3664-3p may be due to the downregulation of *ABCG2, CYP3A4, MCL1,* and *MLH1* in HCT116 cells, and all these genes except *MLH1* in SW480 cells. Additionally, previous studies have demonstrated that miRNAs or other agents targeting resistance-inducing genes can restore sensitivity to chemotherapy drugs by downregulating these genes. *ABCG2*, a well-known gene associated with multidrug resistance, is notably upregulated in glioblastoma, a highly aggressive type of cancer. Early investigations suggest that miRNA-328 directly targets *ABCG2*. Modulating *ABCG2* expression through miRNA-328 in glioblastoma cancer stem cells shows promise as a therapeutic intervention, potentially enhancing the effectiveness of chemotherapy in treating this deadly cancer type [66]. Similarly, overexpression of miRNA-3163 has been found to decrease *ABCG2* expression in retinoblastoma stem cells, leading to increased sensitivity to cisplatin [67].Another study found that miR-548c-3p can reduce *ABCG2* expression, making colorectal cancer cells more responsive to 5-fluorouracil [33]. Mazard et al. demonstrated that sorafenib enhances irinotecan's anti-tumoral activity both in vitro and in vivo, irrespective of *Kras* mutation status. They found that sorafenib improves irinotecan's effectiveness by blocking the drug-efflux pump ABCG2, allowing irinotecan to accumulate inside the cells and increase its toxicity [35]. Liu et al. found that an increase in MCL1 expression, triggered by chemotherapy, plays a key role in chemo-resistance in osteosarcoma. Targeting the miR-375/MCL1 axis could provide a promising approach to enhance the effectiveness of chemotherapy in treating this type of cancer. Additionally, Lu et al. underscored the importance of restoring miR-181b levels to promote apoptosis by reducing both MCL1 and High Mobility Group Box 1 (HMGB1) levels. This strategy made leukemia cells more responsive to varying concentrations of doxorubicin and cytarabine (Ara-C) [68]. Resistance to irinotecan is caused by various mechanisms such as overexpression of ATP-binding cassette (ABC) transporters, inhibition of cell death (apoptosis suppression), anti-senescence effects, changes in drug metabolism, and enhanced DNA repair. However, miR-3664-3p was suggested as a potential means to counteract these resistance mechanisms by targeting the 3′UTR of *ABCG2, CYP3A4*, *MCL1*, and *MLH1* genes. This targeting can increase the sensitivity to irinotecan, making miR-3664-3p a promising candidate for overcoming irinotecan resistance and improving its efficacy. Our study is the first to investigate the effect of miR-3664-3p as a miRNA targeting all four genes on the response of resistant colon cancer cell lines to irinotecan. This approach provides valuable insights into targeting multiple mechanisms of chemo-resistance simultaneously. Concurrently targeting resistant genes may prevent the activation of other resistance mechanisms, thereby helping to overcome resistance to chemotherapy drugs more effectively. Several mechanisms contribute to the resistance to irinotecan, including the overexpression of ATP-binding cassette (ABC) transporters, inhibition of apoptosis, anti-senescence, alterations in drug metabolism, and enhanced DNA repair. This study suggests that miR-3664-3p may counteract these resistance mechanisms by targeting the 3′UTR of *ABCG2, CYP3A4, MCL1,* and *MLH1* genes. This targeting may have increased the sensitivity to irinotecan, making miR-3664-3p a promising candidate for overcoming irinotecan resistance and improving its efficacy. Our study is the first to investigate the effect of miR-3664-3p, which targets all four genes, on the response of resistant colon cancer cell lines to irinotecan. This approach suggests valuable insights into targeting multiple chemo-resistance mechanisms simultaneously.

By targeting resistant genes together, this strategy may help prevent the activation of other resistance mechanisms and more effectively overcome chemotherapy resistance. Given that the PI3K/AKT signaling pathway is shared among the *ABCG2, CYP3A4,* and *MCL1* genes [[21], [22], [23]], inhibiting this pathway by simultaneously targeting its important components may suggest therapeutic applications for overcoming irinotecan chemo-resistance. Since this study is the first to examine the effects of miR-3664-3p on the *ABCG2*, *CYP3A4*, *MCL1*, and *MLH1* genes, additional research is necessary to gain a better understanding of its broader impact and to optimize its applications. Continued studies and ongoing publications in this field will help us gather more comprehensive data to address these aspects in greater detail.

Further research could explore the effect of miR-3664-3p in reducing resistance to other chemotherapy drugs in different types of cancers. Additional studies are necessary to examine the role of this miRNA, along with other miRNAs, in targeting multiple genes involved in resistance simultaneously. Furthermore, assessing the effectiveness of miR-3664-3p in preclinical studies could provide valuable insights into its efficacy and lead to the development of new approaches for sensitizing colorectal cancer tumors to chemotherapeutic drugs, in addition to surgical interventions.

## CRediT authorship contribution statement

**Elham Farrokhnazar:** Writing – original draft, Methodology, Investigation, Formal analysis, Data curation. **Sahar Moghbelinejad:** Supervision, Funding acquisition, Data curation. **Reza Najafipour:** Methodology, Formal analysis. **Ladan Teimoori-Toolabi:** Writing – review & editing, Supervision, Resources, Methodology, Data curation, Conceptualization.

## Ethical consent

No ethical consent was required for this study.

## Availability of data statement

Data will be available upon request.

## Ethics statement

Ethics committee approval was not required for this study as it was conducted entirely through experimental methods without involving animals or human subjects. Additionally, informed consent was unnecessary since no patients were recruited.

## Funding statement

This study was funded by 10.13039/501100006396Qazvin University of Medical Sciences as a Ph.D. student project (Grant Number: 28/20/17104) and 10.13039/501100010679Pasteur Institute of Iran (99/D/290/11476).

## Declaration of competing interest

The authors declare that they have no known competing financial interests or personal relationships that could have appeared to influence the work reported in this paper.
